# Responses to romidepsin by line of therapy in patients with relapsed or refractory peripheral T‐cell lymphoma

**DOI:** 10.1002/cam4.939

**Published:** 2016-12-16

**Authors:** Francine Foss, Barbara Pro, H. Miles Prince, Lubomir Sokol, Dolores Caballero, Steven Horwitz, Bertrand Coiffier

**Affiliations:** ^1^ Yale Cancer CenterNew HavenConnecticut; ^2^ Thomas Jefferson UniversityPhiladelphiaPennsylvania; ^3^ Peter MacCallum Cancer CentreUniversity of MelbourneMelbourne, VictoriaAustralia; ^4^ Moffitt Cancer CenterTampaFlorida; ^5^ Hospital Universitario de SalamancaSalamancaSpain; ^6^ Memorial Sloan Kettering Cancer CenterNew YorkNew York; ^7^ Hospices Civils de LyonLyonFrance

**Keywords:** HDAC inhibitor, peripheral T‐cell lymphoma, refractory, romidepsin

## Abstract

Peripheral T‐cell lymphoma (PTCL) is a heterogeneous group of aggressive non‐Hodgkin lymphomas typically associated with poor prognosis. Most patients with PTCL receive chemotherapy as first‐line treatment, but many experience rapid relapse. For patients with relapsed/refractory PTCL, responses to treatment and long‐term outcomes tend to worsen with increasing lines of therapy. Romidepsin is a potent class I histone deacetylase inhibitor approved by the US Food and Drug Administration for the treatment of PTCL in patients who have received ≥1 prior therapy. A pivotal phase 2 trial of romidepsin in patients with relapsed/refractory PTCL demonstrated an objective response rate of 25% (33/130), including 15% with confirmed/unconfirmed complete response, and a median duration of response of 28 months. In the analysis presented herein, romidepsin was shown to have similar responses and long‐term outcomes in patients with 1, 2, and ≥3 prior lines of treatment, including in patients with disease refractory to the last prior therapy. Although adverse events increased with increasing lines of treatment, the rate of dose modifications and discontinuations due to adverse events was not significantly different. These data support the use of romidepsin as salvage treatment for PTCL irrespective of the number of prior therapies.

## Introduction

Peripheral T‐cell lymphoma (PTCL) is a relatively rare, heterogeneous group of mature T‐cell and natural killer (NK) cell disorders which accounted for 5–10% of the estimated 71,850 new cases of non‐Hodgkin lymphoma (NHL) diagnosed in the United States in 2015 1, 2, 3. Globally, the most common PTCL subtypes are PTCL not otherwise specified (NOS), angioimmunoblastic T‐cell lymphoma (AITL), and anaplastic large‐cell lymphoma (ALCL) 4. Long‐term outcomes for most PTCL subtypes are poor; the 5‐year overall survival (OS) rate was reported at 70% for anaplastic lymphoma kinase (ALK)+ ALCL and <50% for other major subtypes 4, 5.

With the exception of ALK+ ALCL, for which National Comprehensive Cancer Network guidelines suggest first‐line treatment with CHOP (cyclophosphamide, doxorubicin, vincristine, prednisone) or CHOEP (CHOP+etoposide), there is no well‐defined algorithm for treating patients with PTCL 3. Patients with all subtypes typically receive induction chemotherapy as first‐line treatment 3, 6. Anthracycline‐based chemotherapies (e.g., CHOP) are the most common regimens used for the treatment of PTCL, largely based on previous experience and success in the treatment of B‐cell lymphomas 1, 3, 7. Most patients with PTCL respond to chemotherapy, but the responses are typically brief and many patients experience rapid relapse 1, 4. Evidence also suggests that the number of prior lines of therapy should be considered when treatment strategies are evaluated for relapsed or refractory PTCL. A retrospective study (*N* = 205) of patients with PTCL demonstrated that objective response rates (ORRs) and rates of complete response (CR), as well as long‐term outcomes (progression‐free survival [PFS] and OS), worsened with each successive line of treatment 8. Currently, there are limited data in the literature exploring the effect of the number of prior lines of treatment on the efficacy and safety of specific therapies for the treatment of PTCL.

The epigenetic modifying agent romidepsin is a structurally unique, potent, bicyclic class I selective histone deacetylase (HDAC) inhibitor 9, 10, 11 approved by the US Food and Drug Administration (FDA) for the treatment of cutaneous T‐cell lymphoma in patients who had received ≥1 prior systemic therapy and for the treatment of PTCL in patients who had received ≥1 prior therapy 12. A pivotal phase 2, single‐arm, open‐label study of romidepsin in patients with relapsed or refractory PTCL demonstrated an ORR of 25%, including 15% confirmed/unconfirmed CR (CR/CRu) 12, 13, 14. The median duration of response (DOR) was 28 months (median follow‐up, 22.3 months), 14 with the longest response ongoing at 56 months 15. Patients in the study were heavily pretreated, with a median of 2 prior systemic treatments (range: 1–8), and most had advanced disease (70% stage III or IV) 13. Because of generally poorer outcomes with increasing number of lines of treatment for patients with PTCL 8, understanding the clinical profile of romidepsin with regard to the number of previous treatments may be helpful in evaluating treatment strategies for relapsed or refractory PTCL. Here, we present a retrospective analysis of the efficacy and safety of romidepsin in relation to line of treatment in the pivotal phase 2 trial.

## Materials and Methods

### Study design

The details for this phase 2, open‐label, single‐arm, international study (GPI‐06‐0002, NCT00426764) have been reported in detail elsewhere 13. Briefly, eligible patients had one of the following histological subtypes of PTCL 16: PTCL‐NOS, AITL, extranodal NK/TCL nasal type, enteropathy‐type TCL, subcutaneous panniculitis‐like TCL, cutaneous *γδ* TCL, transformed mycosis fungoides, hepatosplenic TCL, ALK− ALCL, or ALK+ ALCL (restricted to patients with relapsed disease following autologous stem cell transplant). Diagnosis of PTCL for enrollment was histologically confirmed by a local pathologist and then reviewed by a central laboratory (Celligent Diagnostics, Charlotte, NC) for PTCL subtyping. Eligible patients had relapsed or refractory disease and had received ≥1 prior systemic therapy, measurable disease according to International Working Group criteria (IWG) 17 and/or measurable cutaneous disease, Eastern Cooperative Oncology Group (ECOG) performance status of 0–2 at enrollment, and adequate bone marrow and organ function (including no known significant cardiac abnormalities). Because hypokalemia and/or hypomagnesemia are known risk factors for cardiac arrhythmia and sudden cardiac death 18, 19, 20, 21 and may be associated with electrocardiogram abnormalities 22, 23, serum potassium and magnesium concentrations were required to be ≥3.8 mmol/L and ≥0.85 mmol/L, respectively. Low levels could be corrected as needed with supplementation to meet inclusion criteria.

Romidepsin was administered intravenously over 4 hours at 14 mg/m^2^ on days 1, 8, and 15 of 28‐day cycles for up to 6 cycles. Patients who had a response (CR/CRu or partial response [PR]) or stable disease (SD) could continue to receive treatment beyond 6 cycles at the discretion of the patient and the investigator. By protocol amendment, patients treated for ≥12 cycles could receive maintenance dosing of 2 rather than 3 doses per 28‐day cycle 14. Patients treated for ≥24 cycles who had received 2 doses per cycle for ≥6 cycles could then be treated at a reduced maintenance dosing of 1 dose per cycle 14. This study was conducted in accordance with the Guidelines of the World Medical Association Declaration of Helsinki in its revised edition (Washington, 2002). The protocol, informed consent forms, and other relevant study documentation were approved by the institutional review boards of all participating institutions. All patients gave written informed consent before study entry.

### Efficacy and safety assessments

Study endpoints have been described in detail elsewhere 13. Briefly, response was assessed every 2 cycles separately by a site investigator and an independent review committee (IRC). IRC assessment was considered primary, and investigator assessments were considered supportive. The primary endpoint of this study was rate of CR/CRu according to 1999 IWG criteria guidelines for NHL response assessment 17 as determined by IRC. Key secondary endpoints included ORR and DOR. DOR was defined as the time from CR/CRu, or PR to disease progression. Time to response, SD, SD for ≥90 days (SD90), OS, and PFS were also assessed. Adverse events (AEs) were documented according to the Medical Dictionary for Regulatory Activities, McLean, VA (version 12.0) and the National Cancer Institute's Common Terminology Criteria for Adverse Events (version 3.0) 13. Drug‐related AEs were defined as those assessed by the investigator as having at least a possible relationship with study medication or those that were missing a relationship assessment. In this retrospective analysis, baseline characteristics, responses to therapy, and safety were examined for patients with 1, 2, or ≥3 lines of prior treatment for PTCL with the objective of investigating the clinical profile of romidepsin in relation to line of treatment 15.

### Statistical methods

All descriptive statistical analyses were performed using the SAS statistical software version 9.2 (SAS Institute, Cary, NC). Differences in response rates were compared using Fisher's exact test. Time to event data was summarized using Kaplan–Meier methods. Differences in DOR, PFS, and OS were compared using the log‐rank test.

## Results

### Patient demographics and baseline characteristics

A total of 131 patients were enrolled; 130 with histopathologically confirmed PTCL were included and one patient diagnosed with diffuse large B‐cell lymphoma was excluded. For this analysis, patients were divided into three groups according to the number of prior systemic treatments (1, 2, and ≥3). There were a comparable number of patients in each group (Table 1). In the group receiving ≥3 prior treatments, 19 patients had 3 lines of prior treatment, 15 had 4 lines, and 14 had ≥5 lines. The overall median age of patients in the study was 61 years (range, 20–83 years) and was comparable across groups. Most patients had an ECOG performance status score of 0 or 1 and an International Prognostic Index score of ≥2. Nearly all patients had received chemotherapy as first‐line treatment. The frequency of previous use of antibody treatments, other immunotherapy, radiation, or autologous stem cell transplant increased as the number of lines of treatment increased. Median time since initial diagnosis also increased with increasing number of lines of treatment.

**Table 1:** Patient demographics and characteristics

	No. of prior systemic therapies		
	1 (*n* = 38)	2 (*n* = 44)	≥3 (*n* = 48)
Male, *n* (%)	30 (79)	27 (61)	31 (65)
Median age (range), years	62.0 (24–83)	61.0 (26–82)	58.5 (20–80)
Geographic location, *n* (%)			
US	22 (58)	17 (39)	21 (44)
Non‐US	16 (42)	27 (61)	27 (56)
ECOG performance status, *n* (%)^a^			
0	13 (34)	22 (50)	11 (23)
1	21 (55)	17 (39)	28 (58)
2	4 (11)	5 (11)	8 (17)
International prognostic index score at study baseline, *n* (%)			
<2	9 (24)	11 (25)	11 (23)
≥2	29 (76)	33 (75)	37 (77)
Median time since diagnosis (range), years	0.8 (0.2–6.4)	1.0 (0.04–6.5)	1.9 (0.1–17.0)
Stage at diagnosis, *n* (%)			
I	4 (11)	8 (18)	6 (13)
II	7 (18)	6 (14)	6 (13)
III	8 (21)	9 (21)	17 (35)
IV	17 (45)	21 (48)	19 (40)
Type of prior systemic therapy, *n* (%)			
Chemotherapy	37 (97)	44 (100)	48 (100)
Monoclonal antibody therapy^b^	4 (11)	5 (11)	11 (23)
Other immunotherapy	0 (0)	3 (7)	11 (23)
Prior autologous stem cell transplant, *n* (%)	0 (0)	3 (7)	18 (38)
Prior radiation therapy, *n* (%)	5 (13)	10 (23)	16 (33)
Refractory to most recent therapy, *n* (%)	12 (32)	20 (46)	17 (35)
PTCL subtype, *n* (%)^c^			
PTCL‐NOS	19 (50)	24 (55)	26 (54)
AITL	9 (24)	10 (23)	8 (17)
ALK− ALCL	6 (16)	6 (14)	9 (19)
Elevated LDH, *n* (%)	18 (47)	24 (55)	28 (58)
Disease in bone marrow, *n* (%)	9 (24)	14 (32)	13 (27)

AITL, angioimmunoblastic T‐cell lymphoma; ALCL, anaplastic large‐cell lymphoma; ALK, anaplastic lymphoma kinase; ECOG, Eastern Cooperative Oncology Group; LDH, lactate dehydrogenase; NOS, not otherwise specified; PTCL, peripheral T‐cell lymphoma; TCL, T‐cell lymphoma.

a One patient had a missing ECOG performance status at baseline.

b Primarily rituximab or alemtuzumab.

c Other subtypes included enteropathy‐type TCL (*n *=* *6), subcutaneous panniculitis‐type TCL (*n* = 3), ALK+ ALCL (*n* = 1), cutaneous *γδ* TCL (*n* = 1), extranodal natural killer/TCL nasal type (*n* = 1), and transformed mycosis fungoides (*n* = 1).

### Efficacy

**Figure 1:**
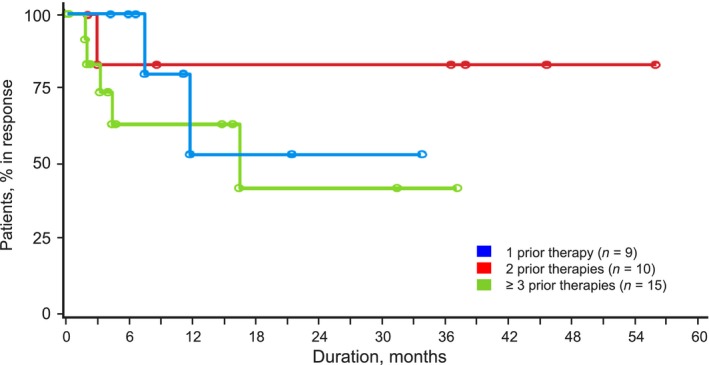
Kaplan–Meier plot of duration of response for patients who had received 1, 2, or ≥3 prior therapies.

**Figure 2:**
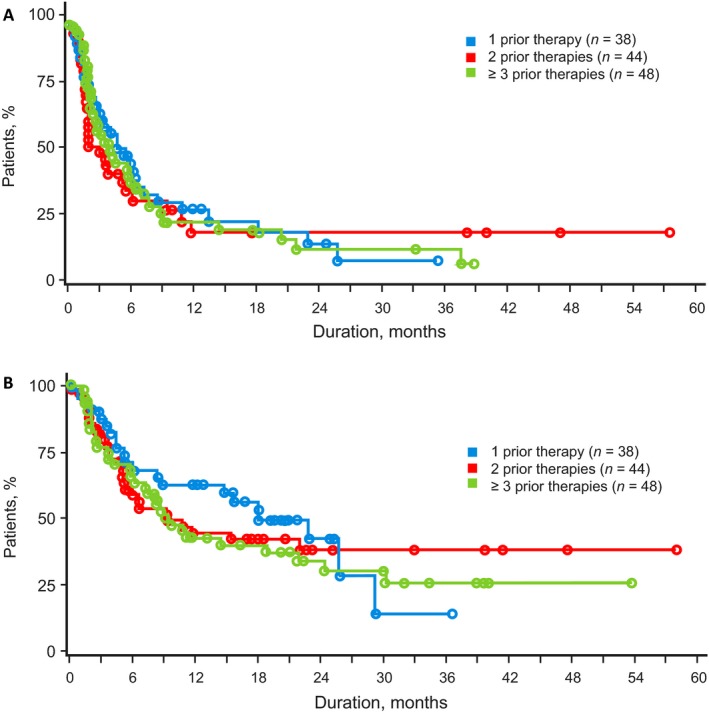
(A) Kaplan–Meier plot of progression‐free survival for patients who had received 1, 2, or ≥3 prior therapies. (B) Kaplan–Meier plot of overall survival for patients who had received 1, 2, or ≥3 prior therapies.

Overall, the responses to romidepsin were similar irrespective of the number of prior lines of treatment. As a benchmark, patients who had received 1 prior systemic therapy (*n *=* *38; Table 2) had an ORR of 24%, median DOR not evaluable (NE), and median PFS and OS of 5.4 and 18.2 months, respectively. There was no statistical difference in response for the primary endpoint (CR/CRu assessed by IRC) or ORR for patients in all three groups (*P* = 0.910 and *P* = 0.634, respectively; Table 2). Rate of SD for patients with 1 prior treatment (34%) was twice that for patients with ≥3 prior treatments (17%), and most patients with a best response of SD achieved SD90 regardless of the number of prior therapies. The median DOR was not statistically different in patients who had received 1, 2, or ≥3 previous lines of treatment (Table 2; Fig. 1) nor were PFS or OS—although median OS for patients who had received 1 prior treatment was twice that of patients who had received 2 or ≥3 prior treatments (Table 2; Fig. 2). For patients with disease refractory to the last prior therapy (*n* = 49), there were also no significant differences in response rates, DOR, and survival by number of prior lines of treatment (Table 3)—the median OS for patients who had received 1 prior treatment was >3 times that for patients who had received 2 or ≥3 previous treatments. For patients who had received transplants (*n* = 21), the ORR was 24%, including 10% with CR/CRu; median DOR was 3.1 months (range, 1.9 to NE), median OS was 8.3 months (range: 2.1 to NE), and median PFS was 2.1 months (range: 1.7–5.6). Most patients (18/21) who had received transplants, including 4 of 5 responding patients who had received transplants, had received ≥3 prior lines of treatment.

**Table 2:** Response rates and long‐term outcomes

	No. of prior systemic therapies			*P* value
	1 (*n* = 38)	2 (*n* = 44)	≥3(*n* = 48)^a^	
ORR, *n* (%)	9 (24)	10 (23)	15 (31)	0.634
CR/CRu	5 (13)	7 (16)	8 (17)	0.910
SD, *n* (%)	13 (34)	11 (25)	8 (17)	
SD90	9 (24)	8 (18)	5 (10)	
DOR, median (range), months	NE (1.9–33.9)	NE (<0.1–56.3)	16.4 (<0.1–37.3)	0.348
PFS, median (range), months	5.4 (0.4–35.5)	3.1 (0.3–57.9)	3.8 (0.3–38.9)	0.907
OS, median (range), months	18.2 (0.4–36.6)	9.4 (0.3–58.1)	9.2 (1.3–53.8)	0.648

CR/CRu, confirmed/unconfirmed complete response; DOR, duration of response; NE, not estimable; ORR, objective response rate; OS, overall survival; PFS, progression‐free survival; SD, stable disease; SD90, SD for ≥90 days.

a *n* = 19 with 3, *n* = 15 with 4, and *n* = 14 with ≥5 prior systemic therapies.

**Table 3:** Response rates and long‐term outcomes for patients with disease refractory to last prior therapy

	No. of prior systemic therapies			*P* value
	1 (*n* = 12)	2 (*n* = 20)	≥3 (*n* = 17)^a^	
ORR, *n* (%)	3 (25)	4 (20)	7 (41)	0.380
CR/CRu	2 (17)	3 (15)	4 (24)	0.894
SD, *n* (%)	4 (33)	2 (10)	0	
SD90	0	2 (10)	0	
DOR, median (range), months	11.6 (7.4–NE)	NE (NE–NE)	16.4 (4.3–NE)	0.264
PFS, median (range), months	5.9 (1.1–13.4)	1.9 (1.4–9.5)	2.0 (1.4–7.7)	0.829
OS, median (range), months	23.0 (4.1–NE)	6.1 (2.1–NE)	7.6 (2.0–21.9)	0.250

CR/CRu, confirmed/unconfirmed complete response; DOR, duration of response; NE, not estimable; ORR, objective response rate; OS, overall survival; PFS, progression‐free survival; SD, stable disease; SD90, SD for ≥90 days.

a *n* = 7 with 3, *n* = 8 with 4, *n* = 1 with 5, and *n* = 1 with 8 prior systemic therapies.

### Safety

**Figure 3:**
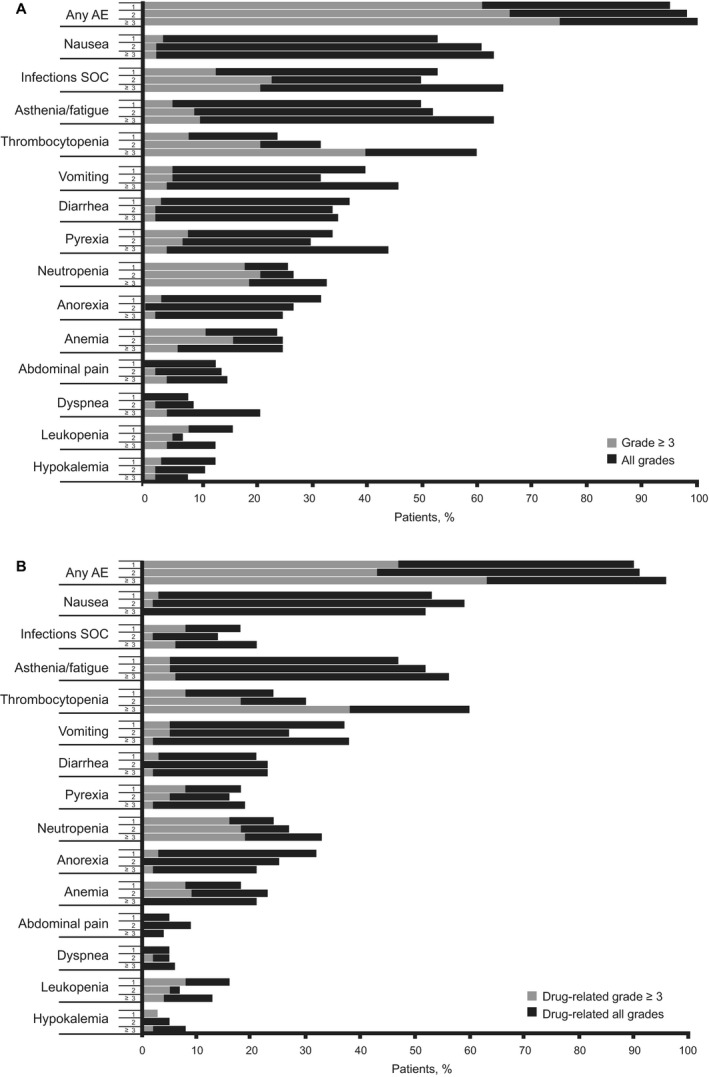
(A) All adverse events (AEs) in patients who had received 1, 2, or ≥3 prior therapies, with grade ≥3 AEs reported in ≥2% of patients overall (*n* = 130). (B) Drug‐related AEs in patients who had received 1, 2, or ≥3 prior therapies, with grade ≥3 AEs reported in ≥2% of patients overall (*n* = 130). SOC, system organ class.

Although the rates of AEs (overall and drug‐related) were similar across groups (Fig.**** 3), rates of overall grade ≥3 AEs were increased with more prior lines of treatment (61%, 66%, and 75% for 1, 2, and ≥3 lines of treatment, respectively). The AE most increased was thrombocytopenia (overall, drug‐related, overall grade ≥3, and drug‐related grade ≥3). For overall AEs, rates of infections (all types pooled), asthenia/fatigue, and dyspnea tended to increase with increasing number of lines of prior treatment; however, trends with infections and dyspnea were not seen for drug‐related AEs, whereas the rate of drug‐related neutropenia did increase with increasing number of prior lines of treatment (24%, 27%, and 33% for all grades; 16%, 18%, and 19% for grade ≥3, respectively).

Rates of dose adjustment (romidepsin dose held or reduced) and discontinuation as a result of AEs were not significantly different by number of prior lines of treatment, although rates were numerically highest for patients who had received ≥3 prior lines of treatment (Table 4). The rates of dose adjustment due to thrombocytopenia in patients who had received 1, 2, and ≥3 prior lines of treatment were 8%, 14%, and 33%, including 8%, 11%, and 31% for drug‐related thrombocytopenia, respectively. Generally, the specific AEs leading to withdrawal were varied, with thrombocytopenia and pneumonia being the most common (each *n* = 3).

**Table 4:** Dose modifications and discontinuations due to adverse events

	No. of prior systemic therapies			*P* value
	1 (*n* = 38)	2 (*n* = 44)	≥3 (*n* = 48)^a^	
Dose held/reduced due to AEs				
Overall	16 (42)	23 (52)	27 (56)	0.446
Drug related	13 (34)	16 (36)	23 (48)	0.368
Discontinuation due to AEs				
Overall	6 (16)	6 (14)	12 (25)	0.384
Drug related	3 (8)	3 (7)	6 (13)	0.696

AE, adverse event.

a *n* = 19 with 3, *n* = 15 with 4, and *n* = 14 with ≥5 prior systemic therapies.

## Discussion

Given the aggressiveness of PTCL, the limited treatment options, and no accepted standard of care, studies that explore the optimal use of current therapies are important to define a robust treatment algorithm. Generally, there have been limited data published on the impact of line of treatment on the clinical efficacy of therapies. A retrospective analysis (*N* = 57) of heavily pretreated patients with relapsed or refractory PTCL treated with pralatrexate demonstrated that a pattern of gradually declining treatment efficacy with number of prior lines of treatment (median PFS of 95 days and 30% ORR at last line of treatment before introduction of pralatrexate) was reversed with pralatrexate (median PFS of 134 days and 40% ORR) 24. This study focused on heavily pretreated patients; of the 57 patients included, 23 had received 3 prior lines of treatment and 34 had received >3 prior lines of treatment before the introduction of pralatrexate. Analyses of patients who had received ≥2 and ≥1 previous lines of treatment in the same study population showed similar trends 25. In addition to pralatrexate and romidepsin, the HDAC inhibitor belinostat and the anti‐CD30 antibody‐drug conjugate brentuximab vedotin have been approved by the US FDA to treat patients with relapsed/refractory PTCL 12, 26, 27, 28. In a phase 2 trial of patients with relapsed/refractory PTCL, belinostat induced an ORR of 26%, including 11% with CR 29. Although no specific analysis was done by line of therapy, ORRs of 25% and 8% were observed for patients who had SD or PD to their prior systemic therapy, respectively. In a phase 2 trial of patients with relapsed/refractory ALCL, brentuximab vedotin demonstrated an ORR of 86%, including 57% with CR 30. Additional agents, including the aurora A kinase inhibitor alisertib and the anti‐CCR4 antibody mogamulizumab, are also being studied in the relapsed/refractory setting 31, 32.

In this study, patients with PTCL treated with romidepsin showed statistically similar ORRs and rates of CR/CRu, as well as DOR, and PFS with 1, 2, or ≥3 prior treatments. Median OS for patients who had received 1 prior treatment was twice that for patients who had received 2 or ≥3 prior treatments (and >3 times when only refractory patients were included). In a recent analysis of a similarly designed National Cancer Institute study of romidepsin in patients with relapsed or refractory PTCL and CTCL, patients with PTCL who had received ≥2 prior treatments had a similar response rate and durability of responses to the overall population 33. The NCI data highlight the versatility of romidepsin in a pretreated population. In addition, although survival data were not collected, the impressive DOR suggested a possible impact on survival 33.

The median DOR for romidepsin in the pivotal study of PTCL was 28 months, with no significant difference in DOR across the three most common PTCL subtypes (PTCL‐NOS, AITL, and ALK− ALCL), and the longest response ongoing at 56 months in a patient with AITL 15, 34. Of patients achieving CR/CRu (19/130), 53% had a DOR of ≥12 months and 32% had a DOR of ≥24 months; 84% (16/19) remained in remission, with a median follow‐up of 25.8 months 14. In addition, achieving SD can be an important therapeutic outcome. Recent reports demonstrated that patients treated with romidepsin who had SD90 or PR as best response had similar long‐term outcomes (OS and PFS) 14, 35, suggesting that even in the absence of an objective response, romidepsin may provide clinical benefit—particularly for patients who were not candidates for transplant. In this study, 34% of patients who had received 1 prior therapy had a best response of SD compared with 17% for patients who had received ≥3 prior therapies (24% vs. 10% for SD90).

The safety profile of romidepsin showed discrete changes with increasing number of lines of treatment. Although differences in the rates of dose modification and withdrawal from treatment due to AEs were not significant, incidences of specific AEs were numerically higher with increasing number of lines of treatment. As romidepsin was introduced in later lines, the incidence of grade ≥3 AEs (overall or drug‐related) tended to increase; most notably, the incidence of thrombocytopenia (overall, drug‐related, overall grade ≥3, and drug‐related grade ≥3), dyspnea (overall all grades and grade ≥3), and drug‐related neutropenia (all grades and grade ≥3) increased. Interestingly, a recent analysis of the AE profile of romidepsin in patients with relapsed or refractory PTCL (from the same pivotal phase 2 study) also showed that patients who received prior monoclonal antibody treatment (primarily rituximab or alemtuzumab) had significantly higher incidence of grade ≥3 infection (20% vs. 4%) and neutropenia (50% vs. 14%) versus those who did not receive antibody treatment 36. These data suggest that fewer severe AEs—hematological toxicities (thrombocytopenia and neutropenia) in particular—may be expected with use of romidepsin in the second line versus later lines of therapy or if sequenced before monoclonal antibody therapy. Although the efficacy of romidepsin was similar in patients who had received 1, 2, or ≥3 prior therapies, the data presented herein suggest that using romidepsin at an earlier line of treatment may be key in optimizing critical aspects of its safety profile. Taken together, the efficacy and safety data support the use of romidepsin irrespective of the number of prior therapies.

## Conflict of Interest

FF: advisory board and consultancy: Celgene Corporation, Eisai, Seattle Genetics, Kyowa Kirin, Spectrum Pharmaceuticals; honoraria: Celgene Corporation, Spectrum Pharmaceuticals; research funding: Celgene Corporation. BP: advisory board and consultancy: Celgene Corporation; honoraria: Celgene Corporation. HMP: advisory board and consultancy: Celgene Corporation; honoraria: Celgene Corporation; research funding: Celgene Corporation. LS: advisory board and consultancy: Celgene Corporation; research funding: Gloucester. SH: advisory board and consultancy: Janssen Pharmaceuticals, Millennium Pharmaceuticals, Amgen, Inc., Bristol‐Myers Squibb, Celgene Corporation; honoraria: Janssen Pharmaceuticals, Millennium Pharmaceuticals, Amgen, Inc., Bristol‐Myers Squibb, Celgene Corporation; research funding: Celgene Corporation, Millennium Pharmaceuticals, Infinity Pharmaceuticals, Kyowa Kirin, Seattle Genetics, Spectrum Pharmaceuticals. BC, advisory board and consultancy: Celgene Corporation. DC has nothing to disclose.
